# Reducing water usage to cool cows by applying smart technologies

**DOI:** 10.3168/jdsc.2025-0827

**Published:** 2025-12-04

**Authors:** L.T. Casarotto, F.X. Amaro, J.M. Lance, D. Onan-Martinez, I.M. Toledo, G.E. Dahl

**Affiliations:** Department of Animal Sciences, IFAS, University of Florida, Gainesville, FL 32611

## Abstract

When trying to achieve optimum production, lactating, and dry dairy cows must be provided with relief from heat stress. The most practical and common methods to alleviate the negative effects of heat and humidity on lactating and dry dairy cows consist of 3 main approaches: shade, natural ventilation, and active cooling, with active cooling being the most advantageous. Our objective was to determine if an automated smart system (Agpro, Paris, TX) for controlling soaker output is as effective as the conventional approach to controlling soakers, which relies on a set frequency after a threshold temperature is reached. Nulliparous (n = 12) and multiparous (n = 30) pregnant Holsteins cows were dried off 40 ± 9 d (multiparous only) before expected calving and randomly assigned to one of 3 treatments in the same barn: 5-min interval (30s on, 5 min off) cooling periods with shade, fans, and soakers (CL; n = 14); Agpro smart cooling with shade, fans, and soakers (SS; n = 14); and heat stress with only shade and natural ventilation and no active cooling system (HT; n = 14) during the dry period until parturition. Daily DMI of individual cows, pen water intake, and pen water usage from soakers were measured daily. Blood samples for hematocrit were taken weekly. Respiration rate (in breaths per minute [bpm]) and rectal temperature (RT) were measured 3 times weekly. Calf birth weight and gestation length were recorded upon parturition. No differences were observed for gestation length, calf birth weight, dry period length, or hematocrit among the treatments. However, HT cows had lower overall DMI than SS (8.6 vs. 10.1 ± 0.4 kg/d; *P* = 0.01) and tended to be lower than CL (8.6 vs. 9.5 ± 0.4 kg/d; *P* = 0.11). Water usage per cow was lowest in the HT group compared with the CL and SS groups (89.6 vs. 225.3 vs. 80.6 ± 156.5 L/cow per day). Respiration rate (68 vs. 53 vs. 48 ± 1.2 bpm; *P* < 0.01), RT (38.8°C vs. 38.4°C vs. 38.3°C ± 0.03°C; *P* < 0.01), and vaginal temperatures (38.9°C vs. 38.6°C vs. 38.7°C ± 0.09°C) were affected by treatment with HT, having the higher values relative to CL and SS, respectively. Compared with conventional cooling, the SS system effectively cooled the animals, decreased the estimated total water usage per cow, and maintained adequate animal welfare.

## Highlights

• Smart soaker cooling reduced water use by >60% compared with CL.
• The SS system cooled cows as effectively as CL.
• Dry matter intake improved with SS and CL versus heat stress.
• Smart soaker cooling supports animal welfare while lowering the water footprint.

## Graphical Abstract

**Figure:**
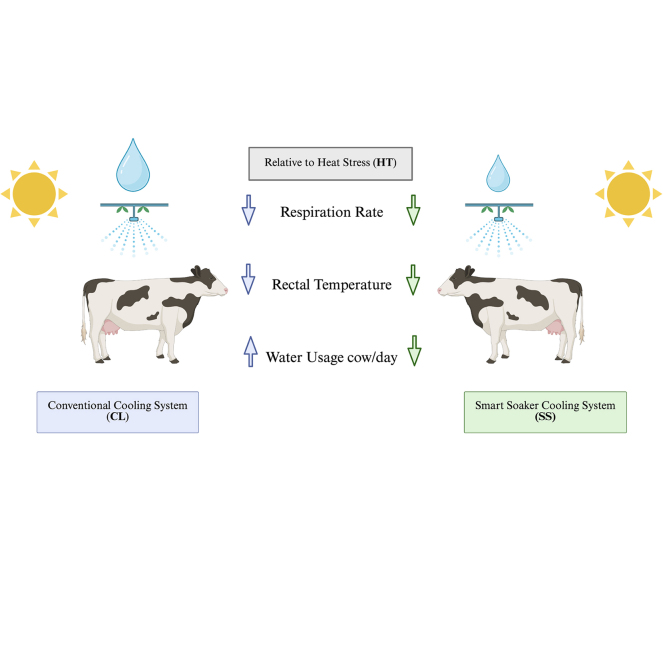
**Summary:** An automated smart soaker (SS) cooling system was evaluated as an alternative to conventional fixed-cycle soakers (CL) for alleviating heat stress in dry, pregnant Holstein cows while reducing water use. Both SS and CL treatments resulted in lower respiration rates and body temperatures, as well as increased dry matter intake, compared with heat-stressed cows without active cooling. Notably, the smart soaker system provided cooling effectiveness equal to that of conventional soakers, while reducing soaker water use by over 60% and lowering total daily water consumption per cow to levels similar to noncooled cows. Reproductive outcomes, calf birth weights, and early-lactation milk yields were unaffected by treatment. Overall, these findings demonstrate that smart soaker technology can maintain animal welfare and thermal comfort during the dry period, while substantially reducing the “blue” water footprint of dairy cooling systems. This makes it a more sustainable solution for heat abatement, particularly in water-limited regions. Graphical abstract created with BioRender (https://app.biorender.com).

The dairy industry faces a growing threat from heat stress, which affects economic viability and animal welfare, as well as water availability for heat abatement. Providing relief from heat and humidity is crucial for lactating and dry dairy cows to achieve optimum production and animal welfare (Collier et al., 2017). To alleviate the negative effects of heat stress, the most practical and effective methods involve shade, ventilation, and evaporative cooling (Collier et al., 2006). These methods can be used individually or in combination and are particularly beneficial in areas such as feed alleys, working areas, and holding pens. Further, it is essential to cool dry cows throughout the entire dry period to minimize the negative impact of heat stress on subsequent milk yield (Fabris et al., 2019). Additionally, effective cooling must be evaluated over a relevant duration rather than being extrapolated from brief periods of cooling.

During heat stress, cows dissipate heat through respiration and sweating, which increases water consumption by up to 50%. Inadequate water supply during heat stress can cause cows to divert water that would normally be used for milk synthesis to physiological processes involved in heat dissipation. To overcome the negative effects of heat stress, producers typically apply evaporative cooling methods that effectively reduce the heat load in hot and humid climates, such as the Southeastern US (Toledo et al., 2019). It is estimated that typical soaker systems use at least 240 L of water per cow each day, and most of the water used is never applied to the cow because the system runs continuously whether or not the cows are under the soakers (Naranjo et al., 2020). Water soaking systems are most effective when combined with adequate air movement along feed bunks and holding areas. This combination promotes the optimum evaporation of water from the skin (Dikmen et al., 2020). The water used to cool cows is considered “blue” water and is the most valuable type because it consists of water from surface or groundwater sources (Naranjo et al., 2020). Thus, there is a tremendous opportunity to reduce the blue water footprint of dairy production by developing smarter systems to more efficiently apply water to cool cows.

This study evaluated whether controlling soaker output with a smart soaker system was as effective as a conventional system—using fixed run cycles based on ambient temperature—in reducing cows' rectal temperature (**RT**) and respiration rate (**RR**; measured in breaths per minute [**bpm**]). We hypothesized that smart soakers would match the cooling effectiveness of the conventional system along with reducing water use.

A research study was conducted over the summer months (July–September) of 2022 at the University of Florida Dairy Unit (Hague, FL). All procedures within the following study were approved by the University of Florida Institutional Animal Care and Use Committee.

The experiment followed a randomized complete block design with 42 pregnant Holsteins cows (30 multiparous and 12 nulliparous). Multiparous cows were dried off 40 ± 9 d before expected calving (multiparous only), and nulliparous cows were included ∼45 d before expected calving (subsequently referred to as the dry period for all cows). Cows were blocked by expected calving date, parity, mature-equivalent milk production, and genomic PTA, and assigned to one of 3 treatments. A limited number of animals were available to be selected in the expected calving date window of this project. All cows were housed and managed in adjacent sand-bedded freestall pens. Treatments were as follows: access to soaking for 1 min at 5-min intervals during the entire dry period with shade, fans (J&D Manufacturing, Eau Claire, WI), and conventional soakers (Rain Bird Manufacturing, Glendale, CA; **CL**, n = 14); smart soaker cooling during the entire dry period with shade, fans, and smart soakers (Agpro, Paris, TX; **SS**, n = 14; Figure 1); and heat stress during the entire dry period (i.e., only shade and natural ventilation until calving; **HT**, n = 14). In the conventional soaker system, when ambient temperature exceeds 21.1°C, the fans operate continuously, and the soakers are activated for 1 min at 5-min intervals.

**Figure 1:**
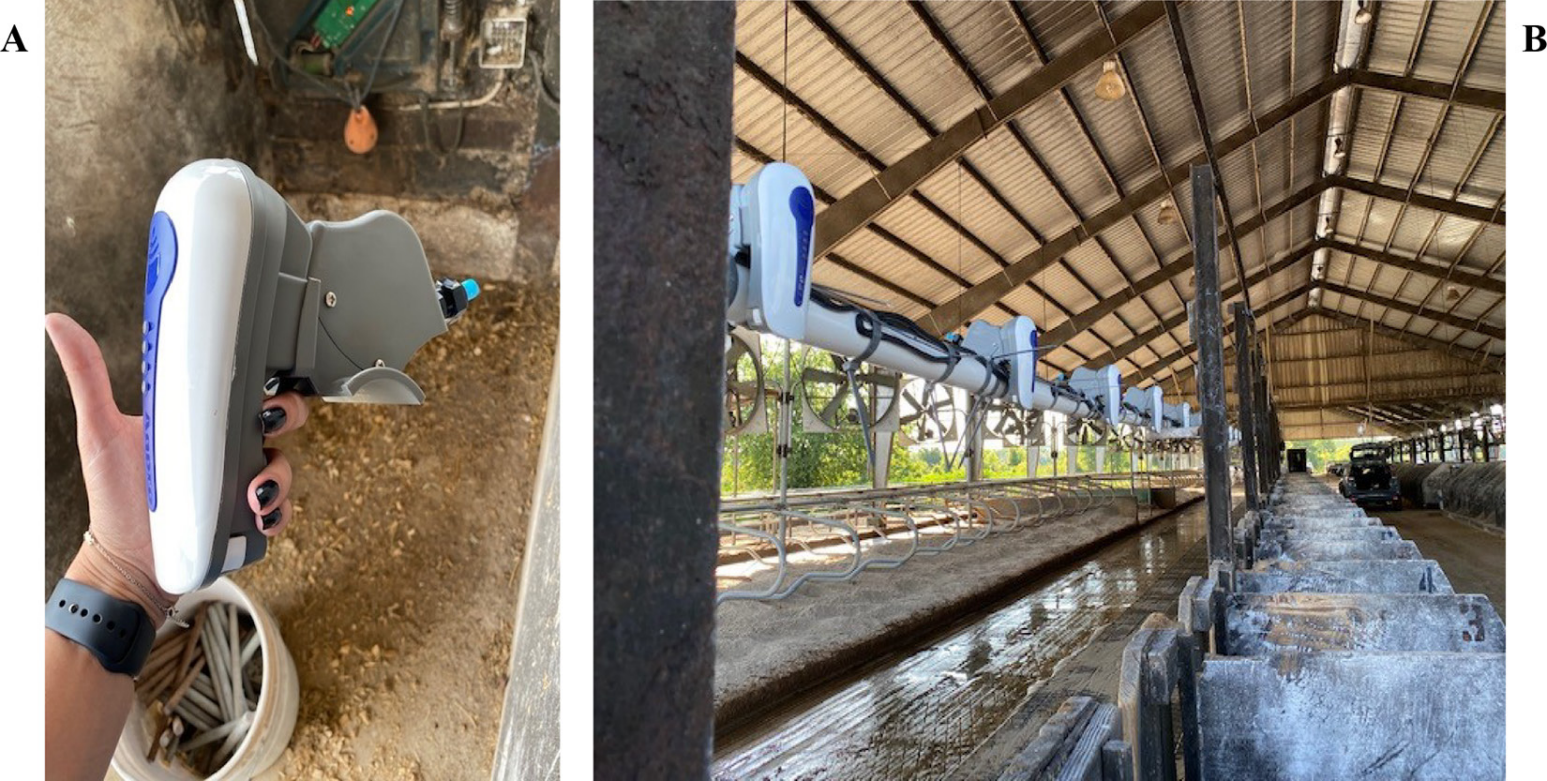
Representative images of an individual SmartSoaker unit (A) and the system installed (B) in the barn where animals were housed for the period of the study (±45 d).

The SS treatment was identical to the CL, except that the soaker system was replaced by the SS units, which detect the presence of a cow under the unit, activate via a sonic signal interruption, and soak the cow for 1 min at 5-min intervals (the same setting as the CL system) for long as the cow is under the unit. Water meters were installed for both CL and SS systems to measure the daily amount of water used by each system in each pen.

All cows were fed a common TMR during the dry period, and the daily DMI of individual cows was recorded during all studies using a Calan gate system (American Calan Inc., Northwood, NH). The total daily water intake for each pen was recorded and individual daily water intake was estimated by dividing water consumption by the number of cows in each pen (L/d). Daily water usage in liters per cow was estimated by adding the water soaker output and the daily water intake for that pen and dividing by the number of cows in that pen.

Blood samples were collected once weekly at 0700 h via coccygeal venipuncture into Vacutainer (Becton Dickinson, Franklin Lakes, NJ) tubes containing sodium heparin anticoagulant from the animals in 3 treatments. After collection, blood samples were immediately placed on ice, and hematocrit was assessed to determine the hydration status of individual cows. Hematocrit was assessed from whole blood from the sodium-heparinized tube. Capillary tubes (Fisher Scientific, Pittsburgh, PA) filled with whole blood were centrifuged at 2,240 × *g* for 3 min at 20°C (room temperature) in a micro hematocrit centrifuge and read with a circular microcapillary hematocrit reader.

Respiration rate and RT were measured thrice weekly at 1200 h for all groups by counting the flank movements for one minute for all the cows in each treatment and by using a rectal thermometer (Genuine Pavia Rectal Veterinary Thermometer, Pavia, Plymouth, MN), respectively, during the whole study. During the first 3 wk of the dry period, the vaginal temperature was recorded using a blank controlled internal drug release (**CIDR**) device containing an iButton thermometer (OnSolution Pty Ltd., Castle Hill, NSW, Australia), which measures temperature at 10 min intervals for up to 7 d. The CIDR were in the schedule of 7 d in use and 7 d rest period, allowing 2 collection of temperature per animal. These thermometers allow for the resolution of mean temperature differences as low as 0.1°C to compare the effectiveness of the cooling systems.

Average daily environmental temperature and relative humidity were accessed using the Florida Automated Weather Network database (https://fawn.ifas.ufl.edu) for the exact coordination of the farm location during the period of the study. The temperature-humidity index (**THI**) was calculated for the barn environment based on the equation recommended by Dikmen and Hansen (2009): THI = (1.8 × T + 32) − [(0.55 − 0.0055 × RH) × (1.8 × T − 26)], where T = ambient temperature (°C) and RH = relative humidity (%).

At parturition, calf birth weight was recorded, and gestation length was calculated by subtracting the breeding date from the calving date. The dry period length was determined by subtraction of the parturition date from the dry-off date. After calving, all cows were housed and managed identically in the same sand-bedded freestall barn with shade and the CL system. Cows were milked twice a day and daily yield, and milk components (protein, fat, and lactose) were measured using the AfiLab milk analyzer (Kibbutz Afikim, Israel) at each milking and SCC were collected and analyzed monthly from DHIA records for their lactation.

All data were analyzed using SAS software (version 9.4; SAS Institute Inc., Cary, NC). Prepartum parameters were analyzed using the GLIMMIX procedure as a generalized linear mixed model. The statistical model included the fixed effects of treatment (SS, CL, and HT), covariate (measurements before treatment started, for RT, RR, and hematocrit), parity (0 to 4), time (days) and calf sex. The random effect of cow nested within treatment was included to account for repeated measures over time, with time specified as the repeated effect. Postpartum parameters, including milk yield and milk composition, were analyzed using the MIXED procedure in SAS. The model included the fixed effects of treatment, genetic PTA (as covariate), time (weeks in milk), and treatment by time interaction. The random effect of cow within treatment was included, and repeated measures were modeled using the covariance structure that best fit the data according to the lowest Akaike information criterion. Water-related variables (animal drinking water, soaker water, and total water usage) were summarized descriptively using the MEANS procedure in SAS. Residual normality and homogeneity of variances were assessed for each continuous dependent variable using the UNIVARIATE procedure in SAS after model fitting. Data are presented as LSM ± SEM unless otherwise indicated. Statistical significance was declared at *P* ≤ 0.05, and a tendency was considered when 0.05 < *P* ≤ 0.15.

During the study period, the environmental temperature of the pens where the animals were housed was 26.1°C ± 1.1°C, relative humidity was 85.7% ± 4.2%, and THI was 77.2 ± 1.3. No differences were found among the SS, CL, and HT groups in dry period length (38.2 vs. 41.3 vs. 39.1 ± 2.3 d; *P* = 0.60), gestation length (273.6 vs. 275.1 vs. 274.3 ± 1.7 d; *P* = 0.80), or calf body weight at birth (38.5 vs. 36.3 vs. 37.4 ± 3.3 kg; *P* = 0.48) for all the animals in the experiment. Hematocrit levels were also not different among treatments during the entire dry period (26.7% vs. 26.6% vs. 26.3% ± 0.40%; *P* = 0.74).

Pregnant dry HT dams had significantly higher RR compared with CL and SS dams (66.4 vs. 52.0 vs. 48.1 ± 1.2 bpm; *P* < 0.01), and RT showed a similar pattern (38.8°C vs. 38.4°C vs. 38.3°C ± 0.03°C; *P* < 0.01). Vaginal temperatures measured with the iButton devices were increased in the HT animals relative to SS and CL cows in the a.m. period (38.7°C vs. 38.5°C vs. 38.6°C ± 0.05°C; *P* < 0.01) and p.m. period (39.1°C vs. 38.7°C vs. 38.8°C ± 0.06°C; *P* < 0.01) for the multiparous cows. Both active cooling systems (CL, and SS) were efficient in keeping cow body temperature in a lower range relative to HT. Dry matter intake was lower in the HT animals when compared with the animals under the CL and SS active cooling systems during the dry period (8.61 vs. 9.48 vs. 10.1 ± 0.40 kg/d; *P* = 0.04).

After parturition, during 10 weeks in milk (**WIM**), no differences were observed in milk yield (35.5 vs. 37.6 vs. 36.6 ± 2.5 kg/d; *P* = 0.84) and milk components such as fat (3.9% vs. 4.1% vs. 4.2% ± 0.25%; *P* = 0.74), protein (4.0% vs. 3.9% vs. 3.7% ± 0.18%; *P* = 0.57) and SCC (3.2 vs. 3.4 vs. 3.7 ± 0.45 × 10^3^ cells/mL; *P* = 0.76) among the multiparous cows previously enrolled in the HT, CL, and SS groups. There was no effect of treatment by time interaction on milk parameters. Moreover, ECM and FCM were similar between treatments. Combining values from both CL and SS treatments did not change the milk yield pattern for the 10 WIM compared with the HT group (37.6 vs. 36.7 ± 7.7 kg/d; *P* = 0.96). A summary of the animal performance is presented in Table 1.

**Table 1:** Least squares means (±SEM) for physiological, reproductive, and productive variables of pregnant Holstein cows exposed to a SmartSoaker cooling system (SS; n = 14), conventional cooling (CL; n = 14), or heat stress (HT; n = 14) during the dry period^1^

Item	Treatment			±SEM	*P*-value^2^	
	SS	CL	HT		TRT	COV
RR, bpm	48.1^a^	52.0^a^	66.4^b^	1.20	<0.001	0.39
RT, °C	38.3^a^	38.4^a^	38.8^b^	0.03	<0.001	0.86
Vaginal temperature, °C						
a.m.	38.6^a^	38.5^a^	38.7^b^	0.05	<0.001	0.89
p.m.	38.8^a^	38.7^a^	39.1^b^	0.06	<0.001	0.99
DMI, kg/d	10.1^a^	9.48^a^	8.61^b^	0.40	0.04	—
Calf BW, kg	38.5	36.3	37.4	3.30	0.48	0.36
GL, d	273.6	275.1	274.3	1.70	0.80	0.99
Dry period, d	38.2	41.3	39.1	2.30	0.60	0.53
Hematocrit, %	26.7	26.6	26.3	0.40	0.74	0.21
Milk yield, kg/d	36.6	37.6	35.5	2.5	0.84	0.12
Fat, %	4.2	4.1	3.9	0.25	0.74	0.13
Protein, %	3.7	3.9	4.0	0.18	0.57	0.89
SCC, × 10^3^ cells/mL	3.7	3.4	3.2	0.45	0.76	—
ECM	42.2	42.8	40.6	3.10	0.87	—
FCM	40.2	40.5	38.0	2.84	0.79	—

a,b Different superscripts within a row indicate differences (*P* ≤ 0.05).

1 Cows were evaluated for respiration rate (RR), rectal temperature (RT), vaginal temperature (iButton thermometer, OnSolution Pty Ltd., Castle Hill, NSW, Australia), DMI, hematocrit, calf birth body weight (BW), gestation length (GL), and dry period length prepartum, and for milk yield and composition (fat, protein, SCC) for 10 WIM. Energy-corrected milk and FCM were calculated.

2 TRT = treatment; COV = covariate.

Animals in the HT group consumed more water daily when compared with the CL and SS animals (89.6 vs. 49.9 vs. 48.8 ± 59.3 L/cow per day). The SS cooling system used less water to cool animals when compared with the CL cooling system (36.1 vs. 184.6 ± 179.4 L/cow per day). Water usage per cow per day was lower in the SS group when compared with CL, but similar in total volume with the HT group (80.6 vs. 225.3 vs. 89.6 ± 156.5 L/cow per day; Figure 2). Data are presented as LSM ± SD.

**Figure 2:**
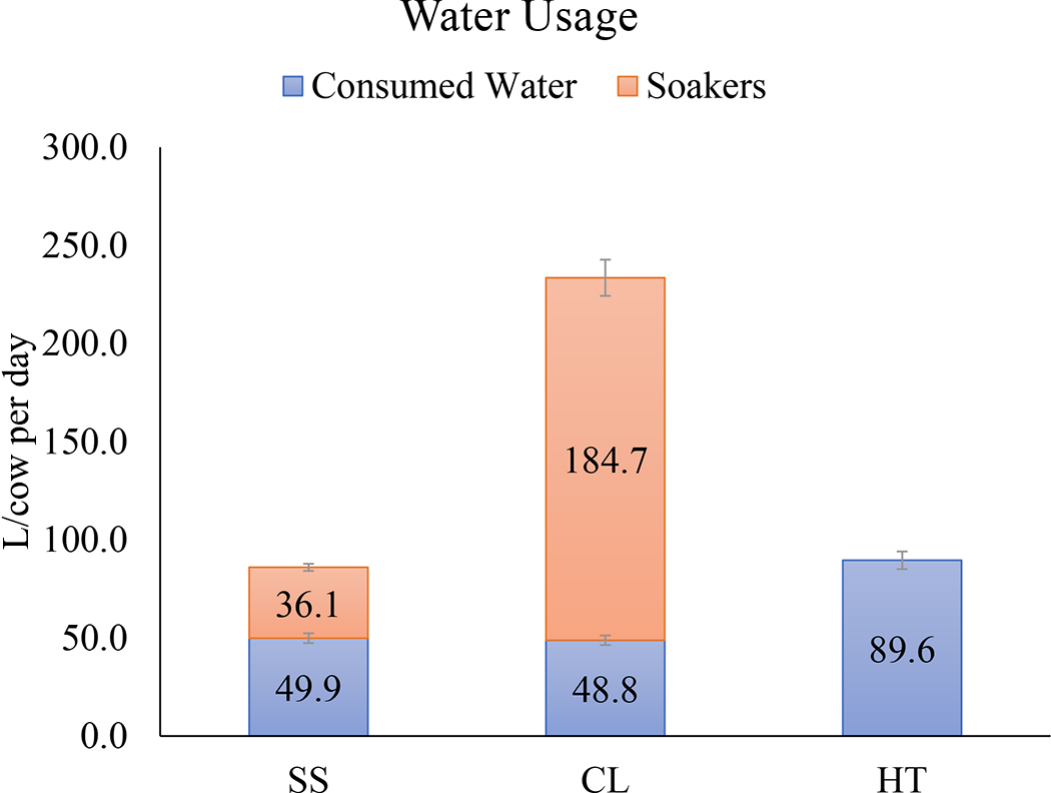
Animal consumed water and soaker output daily (L/cow per day) in the groups with SmartSoaker cooling system (SS; n = 14), traditional active cooling system (CL; n = 14), or heat stress (HT; n = 14) during the dry period. Water usage per cow per day was lower in the SS when compared with the CL but similar in total volume with the HT (80.6 vs. 225.3 vs. 89.6 ± 156.5 L/cow per day). Data presented as LSM ± SD.

The results of this study provide further evidence of the negative consequences of heat stress exposure during late gestation in dairy cows. It is crucial to note that cows that are exposed to THI above 68 during lactation (Zimbelman et al., 2009; Fabris et al., 2019) and 77 during the dry period (Ouellet et al., 2021) experience a rise in RT and RR associated with thermal discomfort and heat stress. In the present study, we observed increased RR and RT in cows exposed to heat stress compared with both conventional and smart cooling systems. Differences between RT and RR in dry cows under cooling versus heat stress systems have been reported in previous studies (Dado-Senn et al., 2021; Ouellet et al., 2021). Moreover, the SS system was found to be equally effective as the CL system in maintaining the animal cooling threshold for RR of 61 bpm, as reported by Toledo et al. (2019). Other studies have reported that heat stress during late gestation can lead to a shorter gestation length (−3 d) and birth weight (−4 kg) compared with cows housed under a conventional cooling system (Monteiro et al., 2014; Laporta et al., 2017). Previous research supports a strong negative correlation between late gestation heat stress and milk yield in the next lactation (Ouellet et al., 2020). Although productive responses were not numerically similar to the current study, it was likely not powered to specifically examine milk yield. Indeed, due to the limited number of animals and different parity stages in this study, we were unable to observe such differences in milk among the treatments despite the RR and RT measures being different, although another production outcome, namely DMI, was improved when animals were cooled. However, a follow-up study with more animals per treatment would likely yield differences in milk production and other variables following late gestation heat stress regardless of the cooling system.

Studies have shown that long-term heat stress affects the ability of dairy cows to regulate their body temperature during the dry period (Fabris et al., 2017). When cows are exposed to heat stress, they are less capable of dissipating the heat generated by their metabolism and digestion (Beede and Collier, 1986). As a result, even dry cows reduce their DMI to compensate for the heat stress (do Amaral et al., 2009; Tao et al., 2011). In this experiment, HT cows showed a decrease in DMI during the dry period relative to CL and SS cows, which is consistent with previous studies that evaluated the effect of heat stress during the dry period (do Amaral et al., 2009; Fabris et al., 2019). Although lower DMI is associated with lower yields in the next lactation (Seyed Almoosavi et al., 2021), suppressed mammary growth during heat stress also leads to reduced yield (Tao et al., 2011; Seyed Almoosavi et al., 2021).

In recent years, there has been a growing focus on reducing the environmental impact of livestock production, particularly regarding water usage (Legesse et al., 2017; Ibidhi and Ben Salem, 2020). To tackle the challenge of water waste in livestock production, innovative cooling technologies are being developed that dramatically reduce water consumption compared with traditional cooling methods for animals (Legesse et al., 2017; Ibidhi and Ben Salem, 2020). The SS system has proven to be highly effective at maintaining optimal body temperatures for cows while substantially lowering water usage each day on a per-cow basis. Previously, farmers have attempted to reduce water use through various means, such as improvements in milking equipment and refining milking processes, with limited success (Naranjo et al., 2020). Sustainable dairy practices that consider the source of water (including blue water) and can reduce the water footprint without negatively affecting productivity or animal welfare are essential for long-term sustainability.

In summary, this study reinforces the detrimental impact of heat stress during late gestation in dairy cows, highlighting the associated risks of increased RT and RR. Exposure to a THI >68 during lactation and >77 during the dry period is linked to significant thermal discomfort and stress in dairy cows. Our findings are consistent with previous research, emphasizing the need to implement effective cooling systems to prevent adverse effects on animal health and productivity. As part of ongoing efforts to mitigate the environmental impact of livestock production, new cooling technologies have been developed to minimize water waste while ensuring effective cooling. Such innovations offer a substantial step forward in reducing the water footprint of dairy production, which is crucial for regions struggling with water scarcity. Although our study demonstrates the efficacy of one of these new cooling systems, further research is necessary under commercial settings and with larger sample sizes and varying stages of parity in order to validate these findings and explore additional benefits. These results underscore the importance of sustainable dairy practices, emphasizing both animal welfare and environmental stewardship as key components in addressing heat stress and water conservation in dairy farming. The successful implementation of more efficient cooling technologies suggests a promising path forward for addressing water scarcity in livestock production while ensuring animal welfare and operational efficiency.
